# Impact of incremental peritoneal dialysis on long-term nutritional trajectories in end-stage kidney disease: a longitudinal cohort study

**DOI:** 10.3389/fnut.2026.1885616

**Published:** 2026-09-17

**Authors:** Yaohua Hu, Xiaohua Zhuang, Jingzhu Zhai, Xinyu Gao, Wenpeng Cui

**Affiliations:** Department of Nephrology, The Second Hospital of Jilin University, Changchun, Jilin, China

**Keywords:** end-stage kidney disease (ESKD), incremental peritoneal dialysis, inverse probability weighting, nutritional trajectories, prognostic nutritional index

## Abstract

**Background:**

Incremental peritoneal dialysis (IPD) seeks to reduce the immediate treatment burden by utilizing residual kidney function. However, its long-term effects on nutritional status remain unclear. This study investigated how an initial IPD strategy influences the longitudinal trajectories of the prognostic nutritional index (PNI) in patients with end-stage kidney disease (ESKD).

**Methods:**

This retrospective study included patients who received peritoneal dialysis (PD) at the Second Hospital of Jilin University between January 2016 and July 2024. We employed linear mixed-effects models to analyze population-level trends in PNI. Group-based trajectory modeling (GBTM) was utilized to identify patterns of PNI evolution, while multivariable Cox models assessed the relationship between these patterns and all-cause mortality. Additionally, multivariable logistic regression, which included multiple imputation and entropy balancing-derived inverse probability weights (IPW), was used to explore the independent association between the type of dialysis and trajectory membership.

**Results:**

A total of 311 patients who initiated PD between 2016 and 2024 were included, of whom 230 received full-dose PD and 81 received IPD. Throughout the follow-up period, 80.25% of patients on IPD were transitioned to full-dose PD at a median of 10 months. Mixed-effects modeling indicated no significant differences in PNI levels or rates of decline between the two modalities (time-by-modality interaction P = 0.378). GBTM revealed three distinct PNI trajectories: low, moderate, and high. The high-level trajectory was associated with a significantly reduced risk of mortality compared to the low-level group (fully adjusted HR = 0.17, P < 0.001). However, in IPW-adjusted models, the initial dialysis modality did not show an independent association with any specific PNI trajectory. These findings remained consistent across multiple sensitivity analyses.

**Conclusions:**

When accompanied by dynamic prescription adjustments, IPD sustains mid- to long-term nutritional trajectories that are comparable to those achieved with full-dose PD.

## 1 Introduction

The rising global burden of end-stage kidney disease (ESKD) requires optimized renal replacement therapies (1–3). Peritoneal dialysis (PD) is a home-based modality that helps preserve residual kidney function (RKF) (4, 5). With the shift toward individualized, goal-oriented dialysis management, incremental peritoneal dialysis (IPD) has gained significant attention as a customized initiation strategy (6, 7). By utilizing preserved RKF, IPD utilizes a lower initial dialysis dose and proactively intensifies the prescription as RKF declines. This approach aims to maintain dialysis adequacy while reducing the initial treatment burden.

While previous evidence indicates that IPD can decrease dialysate consumption and lower the risk of specific dialysis-related complications, concerns regarding its long-term nutritional safety persist (8, 9). Protein-energy wasting (PEW) and malnutrition are prevalent in dialysis populations and are closely associated with cardiovascular events and mortality (10, 11). In PD, ongoing peritoneal protein losses, exposure to glucose-based dialysate, micro-inflammation, and deterioration of RKF create a complex metabolic environment, causing nutritional status to fluctuate dynamically over time (12, 13). A significant clinical concern is that reduced initial clearance may result in early accumulation of uremic toxins, potentially exacerbating anorexia and systemic catabolism. However, much of the existing literature evaluating the nutritional impact of PD has primarily relied on static, single-time-point assessments (14, 15). Cross-sectional methodologies frequently obscure the underlying heterogeneity and dynamic evolution of nutritional status over time, thus failing to accurately capture long-term nutritional trajectories. Furthermore, longitudinal evidence specifically addressing the nutritional impact of IPD strategies remains remarkably scarce.

The Prognostic Nutritional Index (PNI), which combines serum albumin levels and peripheral lymphocyte counts, effectively reflects both protein reserves and immune-inflammatory status. This makes it an ideal biomarker for the longitudinal assessment of nutritional risk in PD patients (16–18). To fill existing methodological gaps, this study employed real-world longitudinal cohort data and utilized advanced linear mixed-effects models along with group-based trajectory models (GBTM). The goal was to systematically compare the long-term evolutionary trajectories of PNI when initiating IPD vs. full-dose PD. Additionally, we explored the associations between different nutritional trajectories and all-cause mortality, aiming to offer real-world evidence that can inform individualized PD prescriptions and proactive nutritional management.

## 2 Methods

### 2.1 Study design and participants

This research was conducted as a longitudinal retrospective cohort study. We enrolled patients diagnosed with ESKD who commenced peritoneal dialysis (PD) for the first time at the Second Hospital of Jilin University between January 1, 2016, and July 31, 2024. The exclusion criteria included: (1) patients who transitioned to PD after undergoing hemodialysis (HD) or kidney transplantation; (2) patients who had fewer than three follow-up laboratory assessments; and (3) patients with incomplete follow-up data. Figure 1 illustrates the detailed patient selection flowchart. Patients were categorized into either the incremental PD (IPD; <8 L/day) group or the full-dose PD (≥8 L/day) group based on their initial PD prescription. All participants were monitored regularly until they either passed away, switched to HD, underwent kidney transplantation, or reached the endpoint of July 31, 2025, whichever occurred first. The study protocol adhered to the ethical principles outlined in the Declaration of Helsinki and received approval from the Ethics Review Committee of the Second Hospital of Jilin University (No. 2025387). Due to the retrospective nature of the study, the necessity for informed consent was waived.

**Figure 1:**
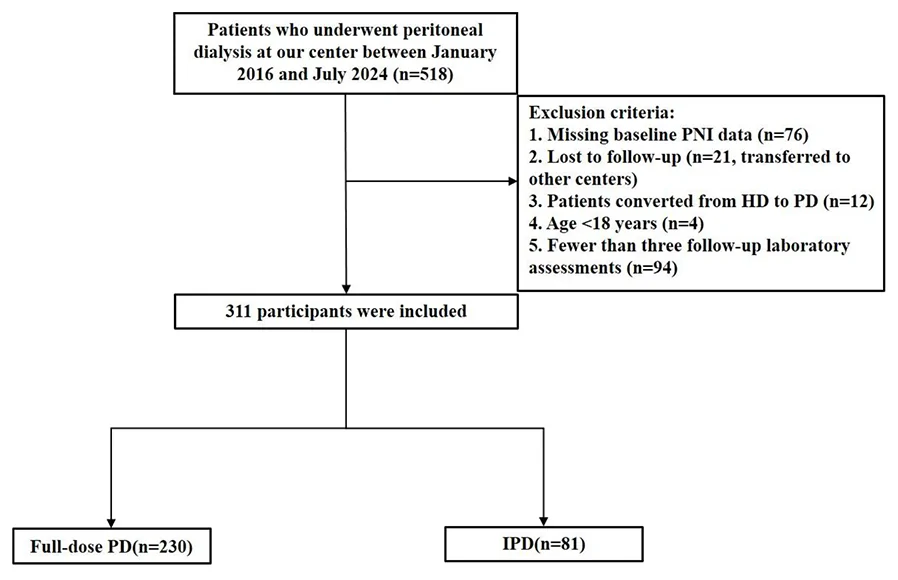
Flowchart of the study population.

### 2.2 Data collection

Data were extracted from the electronic medical records of the Second Hospital of Jilin University. The baseline clinical variables encompassed demographics (age, sex, BMI), blood pressure, lifestyle habits (smoking, alcohol consumption), primary renal disease, and existing comorbidities (diabetes, hypertension, heart disease, stroke). Concurrent medications (ACEIs/ARBs, CCBs, diuretics, α/β-blockers) and peritoneal dialysis (PD) prescriptions, including initial settings and subsequent adjustments, were also documented. Fasting venous blood samples were collected within 2 weeks prior to PD catheterization to establish baseline laboratory indices, which included complete blood counts, liver and kidney function panels (including eGFR), lipid profiles, and fasting glucose levels. To monitor nutritional status over time, serum albumin and peripheral lymphocyte counts were assessed every 3–6 months during the follow-up period.

### 2.3 Definitions of variables and clinical practices

Patients were categorized into the full-dose PD and IPD groups according to their initial PD prescriptions. Given the retrospective observational design of the study, the initial selection of IPD or full-dose PD was not based on a rigid prospective protocol or predefined RKF thresholds. Instead, the initial dialysis prescription was determined by the attending physician based on individualized clinical judgment, taking into account the patient's baseline RKF, uremic symptoms, and volume status. Regarding dialysis prescriptions, full-dose PD was defined as daily PD with a total dialysate volume of ≥8,000 ml/day, typically consisting of four 2-L exchanges per day. IPD was defined as an initial prescription below this threshold, including a total daily dialysate volume of <8,000 ml, fewer than four exchanges per day, or a non-daily treatment schedule (<7 treatment days per week).

Patients receiving PD underwent regular clinical follow-up. The transition from IPD to full-dose PD was determined by the treating nephrologist based on a comprehensive clinical assessment that included dialysis adequacy (e.g., Kt/V), residual urine output, uremic symptoms, and other relevant clinical findings. When reassessment indicated declining solute clearance, reduced residual urine output, or inadequate symptom control, the dialysis prescription was intensified by increasing the dialysate volume, exchange frequency, or both. No single prespecified threshold was used to guide treatment escalation.

The PNI was calculated using the formula: PNI = serum albumin (g/L) + 5 × peripheral blood lymphocyte count (10^9^/L). Baseline PNI was measured within 2 weeks prior to catheterization, with longitudinal assessments conducted every 3–6 months thereafter. To accommodate individual differences in follow-up intervals, the actual follow-up month was used as the time scale for both linear mixed-effects models and group-based trajectory models. The observation period extended from the date of PD catheterization until any occurrence of all-cause mortality, transfer to hemodialysis, kidney transplantation, or the study censor date (July 31, 2025).

### 2.4 Handling of missing variables

The distribution of missing baseline data is summarized in Supplementary Table S1. Specifically, missing values were noted for body mass index (*n* = 39, 12.5%), total Kt/V (*n* = 26, 8.4%), as well as prevalent comorbidities and lifestyle factors (hypertension, diabetes mellitus, stroke, heart disease, smoking, and alcohol consumption; *n* = 16, 5.1% for each), and estimated glomerular filtration rate (*n* = 10, 3.2%). Missing data were handled using multiple imputation by chained equations (MICE) implemented with the *mice* package. Five complete imputed datasets were generated. The imputation models included all variables used in the primary analyses and covariate adjustment to minimize potential imputation bias. Predictive mean matching was used for continuous variables, whereas logistic regression was applied for binary variables. Convergence and model diagnostics were assessed for each imputation model to evaluate the stability of the imputation procedure and the plausibility of the imputed values. The final estimates were obtained by pooling the results across the imputed datasets according to Rubin's rules.

### 2.5 Statistical analysis

Continuous variables are presented as mean ± standard deviation or median (interquartile range) and were compared using Student's *t*-tests or Mann–Whitney *U*-tests, as appropriate. Categorical variables are expressed as frequencies (percentages) and analyzed using Chi-square or Fisher's exact tests.

Longitudinal trajectories of the PNI were evaluated using linear mixed-effects models (*lme4* package) with time (follow-up months), dialysis modality (IPD vs. full-dose PD), and a time-by-modality interaction as fixed effects. Random intercepts and slopes were employed to account for intra-individual variability. The models were adjusted for baseline factors including age, sex, BMI, comorbidities (hypertension, diabetes, heart disease, stroke), lifestyle factors, concurrent medications, eGFR, intact parathyroid hormone levels, and total Kt/V. To capture the heterogeneity in PNI evolution, group-based trajectory modeling (GBTM) (*gbmt* package) was performed. The optimal model specification was selected based on the lowest Bayesian Information Criterion (BIC), average posterior assignment probabilities exceeding 0.80, and maintaining a minimum group proportion of >5% (Supplementary Table S2). Ultimately, a three-group model with linear functional forms provided the best fit and identified three distinct PNI trajectories: high, moderate, and low (Supplementary Table S3). Multivariable Cox proportional hazards models were used to evaluate the associations between PNI trajectories and all-cause mortality, whereas logistic regression models were used to assess the association between dialysis modality and membership in specific trajectory groups. The proportional hazards assumption was evaluated using scaled Schoenfeld residuals and graphical inspection of the residual plots. The global test showed no evidence of violation of the proportional hazards assumption for the overall model. Variable-specific tests were also examined to identify potential deviations from proportionality.

To ensure the robustness of the findings, rigorous sensitivity analyses were conducted. Initially, patients who survived for less than 12 months were excluded, followed by subgroup analyses and testing for interactions. Additionally, multiple imputation was performed to address missing covariates; the resulting datasets were then used to fit both linear mixed-effects and multivariable logistic regression models. Third, entropy balancing was applied separately within each imputed dataset to generate inverse probability weights (IPWs) for estimating the average treatment effect. All baseline covariates were included in the balancing procedure, with exact balance imposed on the specified covariate moments. Positivity was assessed by examining the weight distributions, and adequate covariate balance was defined as an absolute standardized mean difference (ASMD) of <0.1. Finally, weighted logistic regression models were fitted using the *survey* package. The median follow-up time was estimated with the reverse Kaplan–Meier method. To investigate whether preservation of RKF mediated the association between the initial dialysis modality and nutritional outcomes, causal mediation analysis was conducted. To further account for potential confounding by inflammation, high-sensitivity C-reactive protein (hsCRP) was included as an additional covariate. In supplementary sensitivity analyses, the occurrence of PD-associated peritonitis (PDAP) was also included to assess the potential influence of acute inflammatory events on nutritional trajectories. Step-wise multivariable Cox models incorporating both baseline PNI and longitudinal trajectories were constructed to evaluate their independent prognostic values. E-value analyses were also performed to assess the robustness of the primary findings to potential unmeasured confounding. All statistical analyses were conducted using R version 4.4.1, with a two-sided *P*-value <0.05 indicating statistical significance.

## 3 Results

### 3.1 Baseline characteristics

Between January 2016 and July 2024, a total of 518 patients with ESKD undergoing PD were screened. Ultimately, 311 patients met the inclusion criteria and were stratified into the full-dose PD (*n* = 230) and IPD (*n* = 81) groups based on their initial prescriptions. The median follow-up time for the entire cohort, calculated using the reverse Kaplan-Meier method, was 57 months (95% CI: 49–63 months). During the follow-up period, 65 patients (80.25%) in the IPD group required an escalation to full-dose PD at a median of 10 months, whereas 16 patients (19.75%) were able to continue with the IPD regimen.

The mean age of the cohort was 48.70 ± 13.53 years, with 60.45% of the participants identifying as male. The distributions of age and sex were comparable between the two groups (both *P* > 0.05; Table 1). In terms of intergroup differences, the prevalence of diabetes mellitus was significantly higher in the full-dose PD group (41.10 vs. 27.63%, *P* = 0.037). Conversely, the IPD group had a greater proportion of patients with glomerulonephritis as their primary renal disease (63.16 vs. 40.18%, *P* = 0.007) and a higher frequency of ACEI/ARB utilization (35.80 vs. 21.74%, *P* = 0.012). Furthermore, using eGFR as the primary clinical surrogate for RKF, patients in the IPD group showed better preserved RKF, as reflected by a higher eGFR (*P* = 0.008) and lower serum creatinine levels (*P* = 0.004), along with slightly lower alanine aminotransferase levels (*P* = 0.023). All other baseline clinical and laboratory parameters were well-balanced between the two cohorts.

**Table 1:** Baseline clinical characteristics of patients in the two groups.

Variables	Total (*n* = 311)	Full-dose PD (*n* = 230)	IPD (*n* = 81)	*P*
Age (years)	48.70 ± 13.53	49.08 ± 13.37	47.63 ± 13.99	0.408
SBP (mmHg)	144.68 ± 14.81	145.29 ± 14.08	142.88 ± 16.78	0.230
DBP (mmHg)	87.81 ± 10.31	87.78 ± 9.99	87.88 ± 11.28	0.942
Lymphocyte (10^9^/L)	1.24 ± 0.52	1.24 ± 0.52	1.23 ± 0.52	0.915
WBC (10^9^/L)	6.76 ± 2.45	6.72 ± 2.30	6.85 ± 2.87	0.692
Hemoglobin (g/L)	85.58 ± 19.74	84.43 ± 19.76	88.85 ± 19.43	0.083
Albumin (g/L)	35.61 ± 5.56	35.44 ± 5.43	36.09 ± 5.92	0.371
TG (mmol/L)	1.57 (1.14–2.08)	1.60 (1.18-2.10)	1.42 (1.05-1.99)	0.143
TC (mmol/L)	4.55 ± 1.21	4.62 ± 1.25	4.37 ± 1.11	0.152
HDL-C (mmol/L)	1.00 ± 0.31	0.99 ± 0.31	1.04 ± 0.31	0.312
LDL-C (mmol/L)	2.60 ± 0.89	2.65 ± 0.93	2.47 ± 0.74	0.143
BUN (mmol/L)	24.82 (18.39–34.76)	24.94 (18.23-35.68)	24.80 (20.05-31.74)	0.707
Uric acid (μmol/L)	468.88 ± 137.47	471.87 ± 144.09	460.29 ± 116.79	0.517
Serum creatinine (μmol/L)	872.88 ± 368.49	907.95 ± 397.25	767.65 ± 236.64	0.004
eGFR (ml/min/1.73m2)	7.51 (5.42–10.10)	7.21 (5.08-9.82)	8.25 (6.56-10.96)	0.008
BMI (kg/m^2^)	23.61 ± 3.91	23.86 ± 3.90	22.87 ± 3.87	0.068
FBG (mmol/L)	5.81 ± 1.91	5.77 ± 1.86	5.93 ± 2.08	0.536
ALT (u/L)	14.00 (9.00–20.00)	14.00 (9.75–21.00)	12.00 (9.00–16.00)	0.023
AST (u/L)	16.00 (12.00–21.00)	16.00 (12.00–21.00)	15.00 (12.00–19.00)	0.222
Total (Kt/V)	2.09 (1.81–2.55)	2.07 (1.77–2.55)	2.12 (1.88–2.36)	0.608
iPTH (pg/ml)	345.80 (201.90–611.00)	348.50 (197.28–602.23)	317.80 (207.50–646.00)	0.778
Gender, *n* (%)				0.286
Male	188 (60.45%)	135 (58.70%)	53 (65.43%)	
Female	123 (39.55%)	95 (41.30%)	28 (34.57%)	
Hypertension, *n* (%)	275 (93.22%)	207 (94.52%)	68 (89.47%)	0.132
Diabetes, *n* (%)	111 (37.63%)	90 (41.10%)	21 (27.63%)	0.037
Stroke, *n* (%)	42 (14.24%)	32 (14.61%)	10 (13.16%)	0.755
Heart disease, *n* (%)	103 (34.92%)	79 (36.07%)	24 (31.58%)	0.479
Primary renal diseases, *n* (%)				0.007
Glomerulonephritis	136 (46.10%)	88 (40.18%)	48 (63.16%)	
Diabetic nephropathy	91 (30.85%)	75 (34.25%)	16 (21.05%)	
Hypertensive renal disease	38 (12.88%)	31 (14.16%)	7 (9.21%)	
Others	30 (10.17%)	25 (11.42%)	5 (6.58%)	
Smoking, *n* (%)	52 (17.63%)	35 (15.98%)	17 (22.37%)	0.208
Drinking, *n* (%)	27 (9.15%)	17 (7.76%)	10 (13.16%)	0.160
Drug use, *n* (%)				
ARB/ACEI	79 (25.40%)	50 (21.74%)	29 (35.80%)	0.012
CCB	219 (70.42%)	168 (73.04%)	51 (62.96%)	0.087
Diuretic	32 (10.29%)	25 (10.87%)	7 (8.64%)	0.570
α/β blockers	144 (46.30%)	105 (45.65%)	39 (48.15%)	0.698

Percentages for variables are calculated based on valid cases (excluding missing data).

Clinical outcomes during the follow-up period are presented in Supplementary Table S4. Notably, the incidences of PD-associated peritonitis, technique failure requiring transition to hemodialysis, and cause-specific mortality, including cardiovascular and non-cardiovascular mortality, were comparable between the full-dose PD and IPD groups, with no statistically significant differences observed across these endpoints (all *P* > 0.05). To evaluate potential selection bias, baseline characteristics were compared between patients included in the analytic cohort and those who were excluded (Supplementary Table S5). The two groups were generally comparable in terms of demographic characteristics, comorbidities, residual kidney function as assessed by glomerular filtration, and baseline nutritional status.

### 3.2 Longitudinal change in PNI: linear mixed-effects model

Linear mixed-effects modeling was utilized to evaluate the longitudinal trajectories of the PNI (Table 2). Throughout the follow-up period, the entire cohort displayed a statistically significant yet marginal decline in PNI (β = −0.039, *P* = 0.003). However, due to the negligible absolute magnitude of this decrease, the overall nutritional status of the patients remained clinically stable. Modality-specific analysis revealed no significant difference in the overall mean PNI between the IPD and full-dose PD groups (β = −0.504, *P* = 0.537). Furthermore, the time-by-modality interaction term demonstrated that the rate of change in PNI did not differ significantly between the two groups (interaction β = −0.024, *P* = 0.378).

**Table 2:** Linear mixed-effects model evaluating the impact of dialysis modality on longitudinal PNI.

Variable	β (95% CI)	SE	*P*
PNI			
Full-dose PD	0(Reference)	-	-
IPD	−0.504 (−2.110, 1.103)	0.815	0.537
Time, months	−0.039 (−0.064, −0.014)	0.013	0.003
Dialysis modality × time			
Full-dose PD	0 (Reference)	-	-
IPD	−0.024 (−0.077, 0.029)	0.027	0.378

The model was adjusted for baseline covariates, including sex, age, comorbidities (hypertension, diabetes, cardiovascular disease, stroke), smoking, alcohol consumption, eGFR, medication use (ARB/ACEI, calcium channel blockers, diuretics, α/β-blockers), iPTH, total Kt/V, and BMI.

### 3.3 PNI trajectories and their association with all-cause mortality

To uncover the underlying heterogeneity that population-average trends may obscure, GBTM was conducted on the longitudinal PNI data. The analysis identified a three-group model as the optimal fit (BIC = 14211.48; average posterior probabilities > 98% for all groups; see Supplementary Tables S2, S3), which categorized the evolution of PNI into low-, moderate-, and high-level trajectories (Figure 2). Kaplan-Meier analysis revealed significant differences in overall survival across the three trajectory groups (Log-rank *P* < 0.001), with the high-level group showing the highest survival rate (Figure 3). Assessment of multicollinearity indicated that the variance inflation factors (VIFs) for all covariates were below 5 (Supplementary Table S6). Multivariable Cox proportional hazards models confirmed the independent association between PNI trajectories and all-cause mortality (Table 3). When the low-level group was used as a reference, the high-level group had a significantly lower risk of death in Model 1 after adjustment for age and sex (HR = 0.18, 95% CI: 0.10–0.33, *P* < 0.001). This substantial survival advantage persisted through further adjustments for comorbidities, lifestyle factors, and baseline eGFR in Model 2 (HR = 0.20, *P* < 0.001), and remained significant following comprehensive adjustments for BMI, iPTH, total Kt/V, and concurrent medications in Model 3 (*P* < 0.001). The proportional hazards assumption was assessed using scaled Schoenfeld residuals. The global test showed no evidence of an overall violation for the multivariable Cox model (*P* = 0.556). Although the variable-specific test for PNI trajectory suggested a potential departure from proportionality (*P* = 0.047), graphical inspection revealed only modest temporal variation without a clear systematic pattern. Therefore, the Cox model estimates were interpreted with appropriate caution (Supplementary Table S7, Supplementary Figure S1).

**Figure 2:**
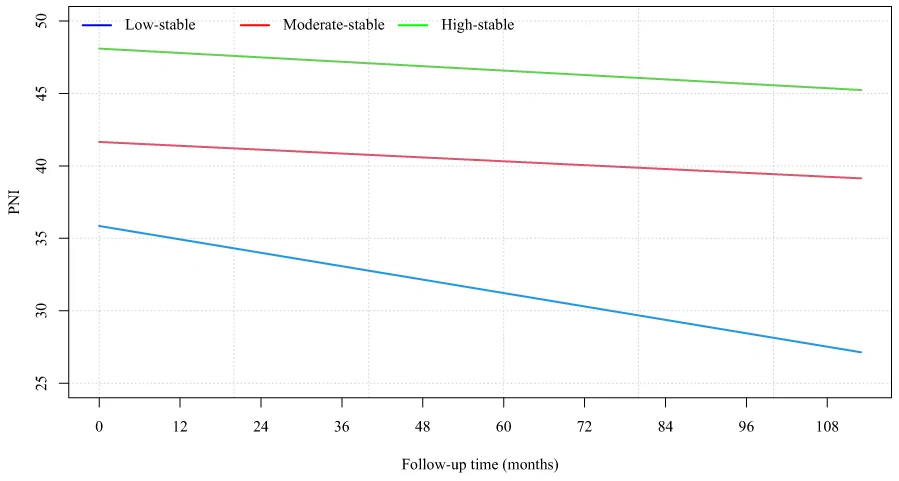
Trajectories of PNI over the follow-up period in patients undergoing peritoneal dialysis.

**Figure 3:**
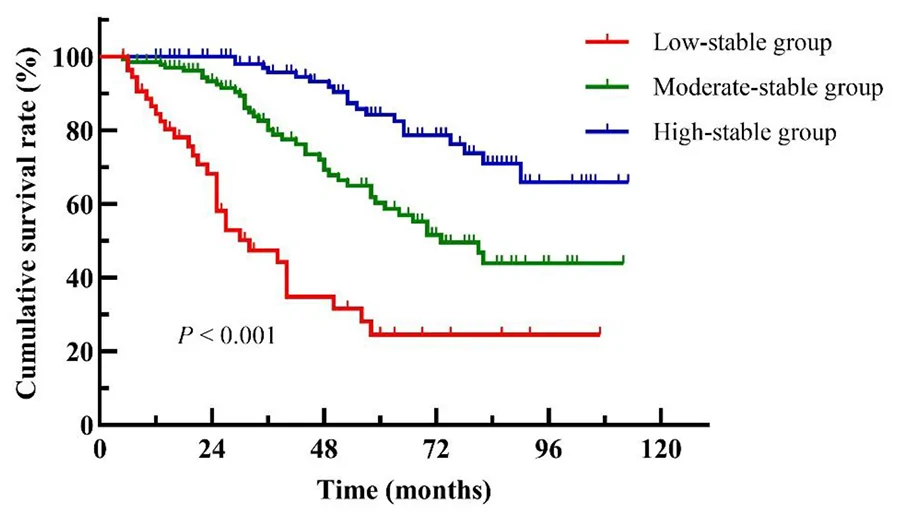
Kaplan-Meier survival curves for all-cause mortality stratified by PNI trajectories.

**Table 3:** Multivariable Cox regression analysis of PNI trajectories and risk of all-cause mortality.

Exposure	Model 1 HR (95%CI)	*P*	Model 2 HR (95%CI)	*P*	Model 3 HR (95%CI)	*P*
Low-stable group	1.00 (ref)		1.00 (ref)		1.00 (ref)	
Moderate-stable group	0.34 (0.21, 0.54)	<0.001	0.39 (0.23, 0.66)	<0.001	0.45 (0.22, 0.92)	0.028
High-stable group	0.18 (0.10, 0.33)	<0.001	0.20 (0.10, 0.38)	<0.001	0.17 (0.07, 0.40)	<0.001

Model 1: adjusted for sex and age; Model 2: model 1 additionally adjusted for comorbidities (hypertension, diabetes, heart disease, stroke), smoking, alcohol consumption, and eGFR; Model 3: model 2 additionally adjusted for medication use (ARB/ACEI, CCB, diuretics, α/β-blockers), iPTH, total Kt/V, and BMI.

### 3.4 Association between dialysis modality and PNI trajectory

Multivariable logistic regression models were developed to further assess the independent association between dialysis modality and specific PNI trajectory membership (Table 4). When membership in the high-level trajectory group was designated as the outcome, no significant difference was observed between the IPD and full-dose PD groups, both in the base model adjusted for age and sex (OR = 0.77, 95% CI: 0.45–1.34, *P* = 0.356) and in the fully adjusted Model 3 (OR = 0.72, 95% CI: 0.36–1.46, *P* = 0.362). Consistently, when evaluating membership in the low-level trajectory group, the fully adjusted model indicated that dialysis modality did not demonstrate a significant independent association (OR = 0.58, *P* = 0.322).

**Table 4:** Logistic regression analysis of the association between the initial PD dialysis modality and the trajectory of PNI.

Exposure	Model 1 OR (95%CI)	*P*	Model 2 OR (95%CI)	*P*	Model 3 OR (95%CI)	*P*
High PNI trajectory						
Full-dose PD	1.00 (ref)	–	1.00 (ref)	–	1.00 (ref)	–
IPD	0.77 (0.45, 1.34)	0.356	0.61 (0.33, 1.12)	0.114	0.72 (0.36, 1.46)	0.362
Low PNI trajectory						
Full-dose PD	1.00 (ref)	–	1.00 (ref)	–	1.00 (ref)	–
IPD	0.51 (0.24, 1.12)	0.096	0.73 (0.32, 1.67)	0.458	0.58 (0.20, 1.71)	0.322

Model 1: adjusted for sex and age.

Model 2: model 1 additionally adjusted for comorbidities (hypertension, diabetes, heart disease, stroke), smoking, alcohol consumption, and eGFR.

Model 3: model 2 additionally adjusted for medication use (ARB/ACEI, CCB, diuretics, α/β-blockers), iPTH, total Kt/V, and BMI.

### 3.5 Sensitivity analysis

To assess the robustness of our primary findings and examine potential effect modifiers, we conducted subgroup analyses stratified by age, sex, hypertension, diabetes mellitus, and baseline renal function. The primary findings were consistent across all subgroups, with no significant interactions observed. This included subgroups defined by baseline renal function, for which the P values for interaction were 0.8714 for the low PNI trajectory and 0.3969 for the high PNI trajectory (Supplementary Table S8). Additionally, to mitigate potential reverse causality, we performed Cox proportional hazards regression after excluding patients who died within the first 12 months of follow-up (Supplementary Table S9). The notable survival advantage associated with the high-level PNI trajectory persisted in the fully adjusted model. To account for the impact of missing baseline covariates, we employed multiple imputation. Re-analysis using linear mixed-effects models and multivariable logistic regression on the imputed datasets yielded results that were highly consistent with our primary analyses (Supplementary Tables S10, S11). Furthermore, to rigorously address potential confounding by indication, we applied entropy balancing to derive IPW on the imputed data. This methodology achieved stringent covariate balance (the covariates included are shown in Supplementary Figure S2), with all absolute standardized mean differences (ASMD) reduced to <0.1 (Supplementary Table S12). Within this rigorously balanced cohort, weighted multivariable logistic regression analysis confirmed that the initial dialysis modality was not independently associated with membership in specific PNI trajectories (Supplementary Table S13). Additionally, causal mediation analysis showed that baseline RKF, represented by eGFR, did not significantly mediate the association between the initial IPD strategy and membership in either the high-level or low-level PNI trajectory group (Supplementary Table S14). In the fully adjusted models, baseline hs-CRP was included following multiple imputation. This additional adjustment did not materially alter either the survival benefit associated with a high-stable PNI trajectory (Supplementary Table S15) or the lack of association between the initial dialysis modality and PNI trajectory membership (Supplementary Table S16). Crucially, additional adjustment for the occurrence of PDAP also did not materially alter the primary effect estimates. The survival advantage associated with the high-stable PNI trajectory remained robust (Supplementary Table S17), and the initial dialysis modality remained unassociated with PNI trajectory membership (Supplementary Table S18). In step-wise analyses (Supplementary Table S19), when baseline PNI and PNI trajectory group were included simultaneously in the model, the high-stable trajectory remained independently associated with improved survival (HR = 0.21, 95% CI: 0.10–0.43, *P* < 0.001), whereas baseline PNI was not significantly associated with survival (HR = 1.00, *P* = 0.897), with no severe multicollinearity (all VIFs <2.5). E-value analyses indicated robustness to unmeasured confounding, requiring an unmeasured confounder with a risk ratio of 6.00 (lower limit: 3.16) associated with both the high-stable trajectory and mortality to fully explain away the survival benefit (Supplementary Table S20). E-values evaluating dialysis modality are reported in Supplementary Table S21.

## 4 Discussion

In this longitudinal cohort study with extended follow-up, we employed advanced analytical techniques, including linear mixed-effects and group-based trajectory models to comprehensively assess the long-term nutritional trajectories, as indicated by the PNI, in patients with ESKD who initiated IPD. Our results reveal that, when coupled with dynamic prescription adjustments based on residual kidney function and dialysis adequacy, both IPD and conventional full-dose PD yield comparable long-term PNI levels and rates of change. Additionally, we identified three distinct PNI evolution trajectories, with the high-level trajectory being independently linked to a significantly lower risk of all-cause mortality. Notably, the initial dialysis modality did not serve as an independent predictor for patient classification within any specific nutritional trajectory, a finding that held steady across rigorous sensitivity analyses.

Previous studies and meta-analyses of IPD have primarily focused on clinical outcomes such as RKF, patient survival, technique survival, and quality of life. In these studies, no significant overall differences were observed in patient survival, peritonitis incidence, or technique survival between IPD and standard-dose peritoneal dialysis (PD). However, the findings regarding RKF preservation were mixed and inconclusive (19–22). In a propensity score–matched cohort of patients undergoing continuous ambulatory peritoneal dialysis (CAPD), IPD was associated with improved survival rates and a slower progression to anuria during the early follow-up period (within the first 6 years); however, this advantage was time-dependent (21). A retrospective study conducted at U.S. dialysis centers revealed that incremental CAPD led to improved outcomes in specific domains of the KDQOL over a one-year period. Notably, this approach did not demonstrate any significant negative impacts on eGFR decline, rates of hospitalization, mortality, or PD discontinuation (23). Additionally, a randomized controlled trial comparing three vs. four daily CAPD exchanges found no significant differences in end-of-follow-up GFR, urine volume, or incidence of anuria. This suggests that an initial reduced prescription does not lead to adverse short-term outcomes (9). Unlike previous studies that concentrated on survival, infection, and quality of life, our research broadens the evaluation to include longitudinal nutritional trajectories. By utilizing longitudinal trajectory analysis, our study reveals a strong correlation between IPD and full-dose PD regarding long-term PNI levels and their slopes. These findings expand the established evidence of the “clinical non-inferiority” of IPD—previously identified in randomized controlled trials and retrospective cohorts—into the domain of nutritional metabolism. In contrast to studies limited to single-point measurements, our trajectory-based approach reveals that nutritional homeostasis can be preserved even during the initial phase of reduced dialysis dosage, thereby addressing clinical concerns about the potential worsening of protein-energy wasting linked to incremental strategies.

The mechanisms behind the neutral effect of IPD on long-term PNI remain unclear, but several explanations are conceivable. One primary rationale for IPD is to enhance the dialysis dose through RKF, which helps prevent early over-dialysis and significant fluid shifts. This strategy may contribute to slowing the deterioration of RKF. Given RKF's crucial role in overall PD adequacy, its preservation is associated with improved volume control and metabolic stability. This, in turn, aids in maintaining nutritional status (6, 24). Second, IPD is typically linked to a lower total dialysate volume and decreased glucose exposure. Elevated glucose levels and their degradation products can induce oxidative stress and inflammation, which can harm the peritoneal membrane and result in metabolic disturbances. Therefore, minimizing the glucose load is viewed as advantageous for alleviating these negative consequences (7, 25, 26). However, the potential biological advantages may be diminished by the lower initial peritoneal clearance of small solutes characteristic of the IPD regimen. Although RKF offers some compensation, the reduced dialysis dose at the outset could theoretically result in a temporary buildup of metabolic toxins. This early accumulation of uremic toxins could theoretically suppress appetite, exacerbate systemic inflammation, and trigger protein catabolism (27). Crucially, the long-term stability of the PNI trajectory must be understood within the framework of dynamic prescription management. In our cohort, 80.25% of patients in the IPD group experienced a timely transition to full-dose PD, with a median time of 10 months, to accommodate changing metabolic needs as RKF declined. This targeted intensification likely addressed the initial dialysis dose deficit.

Our study possesses several significant strengths. First, unlike traditional single-time-point cross-sectional analyses, we utilized linear mixed-effects models and GBTM to dynamically analyze years of longitudinal, repeatedly measured data. This methodology effectively captures the underlying evolutionary patterns of long-term nutritional status in patients undergoing peritoneal dialysis. Second, the comprehensive application of advanced statistical techniques—specifically, multiple imputation and entropy balancing-derived IPW—substantially reduced potential confounding by indication, thereby enhancing the robustness of our findings. Third, our cohort mirrors real-world clinical practice, providing valuable real-world evidence suggesting that, with strict monitoring and adaptive prescription adjustments, IPD can mitigate the early treatment burden while maintaining long-term nutritional safety. IPD may represent a viable initial treatment strategy for patients initiating dialysis who have relatively well-preserved RKF. However, its safe implementation requires regular nutritional assessment and ongoing clinical monitoring. If dialysis adequacy, as reflected by Kt/V, declines, residual urine output decreases, or uremic symptoms develop, clinicians should promptly intensify the dialysis prescription to full-dose PD. While GBTM trajectories are inherently influenced by baseline values, our sensitivity analyses suggest that longitudinal PNI trajectory membership provides prognostic information beyond that provided by a single baseline PNI measurement, even after adjustment for baseline PNI.

However, several limitations should be acknowledged. First, the single-center retrospective design inevitably introduced the potential for residual confounding and selection bias, limiting our ability to establish a causal relationship between the initial dialysis modality and the observed nutritional trajectories, despite rigorous statistical adjustments such as IPW. Second, the absence of detailed information on specific dietary prescriptions, including exact daily protein and caloric intake, and nutritional supplementation limited our ability to explore the mechanisms underlying variation in nutritional trajectories. Consequently, we could not determine the extent to which unmeasured proactive nutritional management contributed to the comparable nutritional outcomes observed between the two dialysis modalities. Third, the lack of systematically collected data on non-fatal hospitalizations occurring at external facilities precluded a rigorous comparison of the overall morbidity burden between the two modalities. Fourth, nutritional status was assessed exclusively using the PNI. Although the PNI is a validated biochemical and immune-inflammatory biomarker, the retrospective design precluded complementary multi-dimensional evaluations, such as subjective scoring (e.g., SGA), bioimpedance, and morphofunctional assessments (e.g., muscle ultrasound). This reliance on a single marker limits the comprehensiveness of our conclusions regarding nutritional safety. Fifth, our cohort predominantly consisted of patients undergoing CAPD. Given the differences in solute clearance kinetics, dwell times, and glucose absorption between CAPD and automated peritoneal dialysis (APD), our findings may not be directly generalizable to patients receiving APD. To definitively validate these findings and overcome current observational constraints, future randomized controlled trials comparing IPD and full-dose PD strategies are warranted. Such trials should incorporate serial morphofunctional nutritional assessments, including muscle ultrasound and bioimpedance analysis, to elucidate the multidimensional metabolic impact of incremental strategies.

## 5 Conclusion

This longitudinal cohort study demonstrates that an initial IPD strategy sustains mid- to long-term nutritional trajectories comparable to those of conventional full-dose peritoneal dialysis. While maintaining a high-level nutritional trajectory is independently associated with a significantly lower risk of all-cause mortality, the initial dialysis modality (IPD vs. full-dose) does not serve as an independent determinant of a patient's likelihood of falling into unfavorable nutritional trajectories. Thus, for patients with ESKD and preserved residual kidney function, IPD emerges as a safe and feasible individualized initiation strategy that effectively alleviates the early treatment burden without compromising long-term nutritional safety.

## Data Availability

The raw data supporting the conclusions of this article will be made available by the authors, without undue reservation.
