# Supplementary material for: Impact of incremental peritoneal dialysis on long-term nutritional trajectories in end-stage kidney disease: a longitudinal cohort study
Source: Front Nutr. 2026 Sep 17;13:1885616. doi: 10.3389/fnut.2026.1885616 (PMC13627045; doi:10.3389/fnut.2026.1885616)
Supplement: Supplementary file 1 [file Table_1.docx]

**Supplementary Material**

**Table S1. Missing data of the study participants**

**Table S2. Selection of the optimal trajectory model**

**Table S3. Parameter estimates for the PNI trajectory model**

**Table S4**. Clinical outcomes of patients during the follow-up period

**Table S5.** Comprehensive comparison of baseline characteristics between the analyzed cohort and excluded patients

**Table S6. Variance inflation factors (VIF) for collinearity screening of covariates**

**Table S7.** Proportional hazards assumption test for the multivariable Cox regression model

**Table S8. Subgroup analyses and tests for interaction**

**Table S9.** Sensitivity analysis of the association between PNI trajectories and risk of all-cause mortality, excluding patients who died within the first 12 months.

**Table S10.** Linear mixed-effects model for longitudinal PNI after multiple imputation for missing data.

**Table S11. Sensitivity analysis of the association between initial dialysis modality and PNI trajectory membership using multiple imputation**

**Table S12.** Covariate balance before and after entropy balancing-derived inverse probability weighting

**Table S13. Sensitivity analysis of the association between initial dialysis modality and PNI trajectory membership using inverse probability weighted multivariable logistic regression with multiple imputation.**

**Table S14. Causal mediation analysis of baseline RKF (eGFR) on the association between initial dialysis modality and PNI trajectories**

**Table S15.** Sensitivity analysis of the association between PNI trajectories and risk of all-cause mortality adjusting for baseline CRP

**Table S16.** Sensitivity analysis of the association between initial dialysis modality and PNI trajectory membership adjusting for baseline CRP

**Table S17.** Sensitivity analysis of the association between PNI trajectories and all-cause mortality, additionally adjusted for PDAP

**Table S18.** Sensitivity analysis of the association between initial PD modality and PNI trajectories, additionally adjusted for PDAP

**Table S19.** Step-wise sensitivity analysis evaluating the prognostic value of baseline PNI and longitudinal PNI trajectories for all-cause mortality

**Table S20.** E-value analysis to assess the robustness of the association between PNI trajectories and all-cause mortality to unmeasured confounding

**Table S21.** E-value analysis to assess the robustness of the association between initial dialysis modality and PNI trajectory membership to unmeasured confounding

**Figure S1.** Scaled Schoenfeld residual plot for the PNI trajectory.

**Figure S2.** Covariate balance before and after inverse probability weighting.

**Table S1. Missing data of the study participants**

| **Variable** | **Number of missing values** | **Missing rate (%)** |
| --- | --- | --- |
| BMI | 39 | 12.5 |
| Total Kt/V | 26 | 8.4 |
| Hypertension | 16 | 5.1 |
| Diabetes mellitus | 16 | 5.1 |
| Stroke | 16 | 5.1 |
| Heart disease | 16 | 5.1 |
| Smoking status | 16 | 5.1 |
| Drinking status | 16 | 5.1 |
| Primary renal diseases | 16 | 5.1 |
| eGFR | 10 | 3.2 |

**Table S2. Selection of the optimal trajectory model**

| **Number of trajectory groups** | **Polynomial order by group** | **BIC** | **Proportion in each group (%)** | **Mean posterior probability (%)** |
| --- | --- | --- | --- | --- |
| 1 | 1 | 14803.21 | 100 | 100 |
| 2 | 1/1 | 14340.35 | 67.37/32.63 | 99.88/99.63 |
| 3 | 1/1/1 | 14211.48 | 17.96/43.11/38.92 | 99.54/98.77/99.71 |
| 4 | 1/1/1/1 | 14240.72 | 41.32/38.32/11.98/8.38 | 99.08/99.48/97.15/99.99 |
| 5 | 1/1/1/1 | 14252.66 | 37.42/38.32/12.28/NA/11.98 | 99.49/98.86/98.92/NA/99.58 |

**Table S3. Parameter estimates for the PNI trajectory model**

| **Trajectory group** | **Trajectory pattern** | **Parameter** | **Estimate** | **Standard error** | **t value** | **P value** |
| --- | --- | --- | --- | --- | --- | --- |
| 1 | Low-stable group | Intercept | 35.9299 | 0.1714 | 209.650 | <0.001 |
|  |  | Linear | -0.0771 | 0.0068 | -11.42 | <0.001 |
| 2 | Moderate-stable group | Intercept | 41.6738 | 0.1407 | 296.292 | <0.001 |
|  |  | Linear | -0.0222 | 0.0048 | -4.672 | <0.001 |
| 3 | High-stable group | Intercept | 48.1202 | 0.1726 | 278.753 | <0.001 |
|  |  | Linear | -0.0253 | 0.0050 | -5.047 | <0.001 |

**Table S4**. Clinical outcomes of patients during the follow-up period

| **Outcomes** | **Total (n = 311)** | **Full-dose PD (n = 230)** | **IPD (n = 81)** | **P value** |
| --- | --- | --- | --- | --- |
| **PD-associated peritonitis**, n (%) | 49 (15.8%) | 40 (17.4%) | 9 (11.1%) | 0.182 |
| **Clinical status at the end of follow-up**, n (%) |  |  |  | 0.075 |
| Continue PD | 142 (45.7%) | 97 (42.2%) | 45 (55.6%) |  |
| Death | 91 (29.3%) | 69 (30.0%) | 22 (27.2%) |  |
| Transfer to hemodialysis | 78 (25.1%) | 64 (27.8%) | 14 (17.3%) |  |
| **Cause of death**, n (%) |  |  |  | 0.871 |
| Cardiovascular death | 40 (44.0%) | 30 (43.5%) | 10 (45.5%) |  |
| Other causes | 51 (56.0%) | 39 (56.5%) | 12 (54.5%) |  |

Notes: Percentages for the cause of death are calculated based on the total number of deaths in each respective group

**Table S5.** Comprehensive comparison of baseline characteristics between the analyzed cohort and excluded patients

| **Variables** | **Total Screened (n = 518)** | **Excluded Patients (n = 207)** | **Analyzed Cohort (n = 311)** | **P value** |
| --- | --- | --- | --- | --- |
| **Sex**, n (%) |  |  |  | 0.194 |
| Male | 322 (62.3%) | 134 (65.0%) | 188 (60.5%) |  |
| Female | 194 (37.5%) | 71 (34.5%) | 123 (39.5%) |  |
| Missing | 1 (0.2%) | 1 (0.5%) | 0 (0%) |  |
| **Age** (years) | 49.1 ± 14.3 | 49.7 ± 15.4 | 48.7 ± 13.6 | 0.414 |
| **Hypertension**, n (%) | 436 (91.8%) | 161 (89.4%) | 275 (93.2%) | 0.146 |
| **Diabetes mellitus**, n (%) | 179 (37.7%) | 68 (37.8%) | 111 (37.6%) | 0.974 |
| **Stroke**, n (%) | 64 (13.5%) | 22 (12.2%) | 42 (14.2%) | 0.533 |
| **Heart disease**, n (%) | 165 (34.7%) | 62 (34.4%) | 103 (34.9%) | 0.917 |
| **Primary renal disease**, n (%) |  |  |  | 0.233 |
| Glomerulonephritis | 225 (47.4%) | 89 (49.4%) | 136 (46.1%) |  |
| Diabetic nephropathy | 144 (30.3%) | 53 (29.4%) | 91 (30.8%) |  |
| Hypertensive renal disease | 52 (10.9%) | 14 (7.8%) | 38 (12.9%) |  |
| Others | 53 (11.2%) | 23 (12.8%) | 30 (10.2%) |  |
| Missing | 1 (0.2%) | 1 (0.6%) | 0 (0%) |  |
| **Smoking**, n (%) | 90 (18.9%) | 38 (21.1%) | 52 (17.6%) | 0.347 |
| **Drinking**, n (%) | 52 (10.9%) | 25 (13.9%) | 27 (9.2%) | 0.109 |
| **BMI** (kg/m²), Mean ± SD | 23.8 ± 4.3 | 23.8 ± 3.8 | 23.8 ± 4.6 | 0.979 |
| **SBP** (mmHg), Mean ± SD | 143.6 ± 16.2 | 141.9 ± 18.1 | 144.7 ± 14.8 | 0.069 |
| **DBP** (mmHg), Mean ± SD | 87.2 ± 11.1 | 86.2 ± 12.3 | 87.8 ± 10.3 | 0.114 |
| **ARB/ACEI use**, n (%) | 130 (25.1%) | 51 (24.6%) | 79 (25.4%) | 0.844 |
| **CCB use**, n (%) | 334 (64.5%) | 115 (55.6%) | 219 (70.4%) | <0.001 |
| **Diuretic use**, n (%) | 53 (10.2%) | 21 (10.1%) | 32 (10.3%) | 0.958 |
| **α/β-blockers use**, n (%) | 227 (43.8%) | 83 (40.1%) | 144 (46.3%) | 0.163 |
| **WBC** (10^9/L) | 7.0 ± 4.4 | 7.5 ± 6.9 | 6.8 ± 2.5 | 0.086 |
| **Hemoglobin** (g/L) | 86.7 ± 20.3 | 89.2 ± 21.4 | 85.6 ± 19.7 | 0.076 |
| **Albumin** (g/L) | 35.6 ± 5.8 | 35.5 ± 6.2 | 35.6 ± 5.6 | 0.838 |
| **TG** (mmol/L) | 1.8 ± 1.1 | 1.8 ± 1.2 | 1.8 ± 1.0 | 0.778 |
| **TC** (mmol/L) | 4.5 ± 1.2 | 4.4 ± 1.3 | 4.6 ± 1.2 | 0.365 |
| **HDL-C** (mmol/L) | 1.0 ± 0.3 | 1.0 ± 0.4 | 1.0 ± 0.3 | 0.828 |
| **LDL-C** (mmol/L) | 2.6 ± 1.2 | 2.6 ± 1.8 | 2.6 ± 0.9 | 0.805 |
| **BUN** (mmol/L) | 25.2 (18.2, 35.3) | 26.2 (17.5, 36.1) | 24.8 (18.4, 34.8) | 0.644 |
| **Uric acid** (µmol/L) | 471.1 ± 141.3 | 475.6 ± 149.1 | 468.9 ± 137.5 | 0.630 |
| **Serum creatinine** (µmol/L) | 876.4 ± 396.9 | 883.7 ± 450.7 | 872.9 ± 368.5 | 0.788 |
| **eGFR** (ml/min/1.73m²) | 5.9 (4.4, 7.9) | 5.9 (4.1, 8.4) | 5.9 (4.5, 7.6) | 0.603 |
| **Fasting glucose** (mmol/L) | 6.0 ± 2.3 | 6.5 ± 3.0 | 5.8 ± 1.9 | 0.003 |
| **ALT** (U/L) | 13.0 (9.0, 20.0) | 12.0 (9.0, 19.5) | 14.0 (9.0, 20.0) | 0.187 |
| **AST** (U/L) | 18.3 ± 11.4 | 18.1 ± 12.3 | 18.4 ± 11.0 | 0.815 |
| **iPTH** (pg/mL) | 351.0 (202.1, 602.2) | 323.3 (156.3, 514.9) | 375.3 (217.7, 618.6) | 0.030 |

**Table S6. Variance inflation factors (VIF) for collinearity screening of covariates**

| **Variable** | **VIF** |
| --- | --- |
| Nutritional trajectory group | 1.2 |
| Sex | 1.4 |
| Age | 1.3 |
| Hypertension | 1.1 |
| Diabetes mellitus | 1.6 |
| Stroke | 1.2 |
| Heart disease | 1.1 |
| Smoking status | 1.4 |
| Drinking status | 1.4 |
| eGFR | 1.3 |
| ACEI/ARB | 1.0 |
| CCB | 1.2 |
| Diuretics | 1.1 |
| Beta-blockers | 1.2 |
| BMI | 1.6 |
| iPTH | 1.2 |
| Total Kt/V | 1.4 |

**Table S7.** Proportional hazards assumption test for the multivariable Cox regression model

| **Covariates** | **Chi-square** | **df** | **P value** |
| --- | --- | --- | --- |
| PNI trajectory | 6.118 | 2 | 0.047 |
| Age | 0.451 | 1 | 0.502 |
| Sex | 0.001 | 1 | 0.971 |
| Hypertension | 3.068 | 1 | 0.080 |
| Diabetes mellitus | 0.184 | 1 | 0.668 |
| Stroke | 0.575 | 1 | 0.448 |
| Heart disease | 1.387 | 1 | 0.239 |
| Smoking status | 0.469 | 1 | 0.494 |
| Alcohol consumption | 0.165 | 1 | 0.685 |
| eGFR | 0.311 | 1 | 0.577 |
| ACEI/ARB | 1.276 | 1 | 0.259 |
| CCB | 0.949 | 1 | 0.330 |
| Diuretics | 0.000 | 1 | 0.998 |
| α/β-blockers | 0.167 | 1 | 0.683 |
| BMI | 1.819 | 1 | 0.177 |
| iPTH | 0.063 | 1 | 0.802 |
| Total Kt/V | 0.022 | 1 | 0.882 |
| hs-CRP | 1.884 | 1 | 0.170 |
| Global test | 17.507 | 19 | 0.556 |

Notes: The proportional hazards (PH) assumption was formally evaluated using scaled Schoenfeld residuals with Kaplan-Meier time transformation. A global test P value > 0.05 indicates that the overall model robustly satisfies the PH assumption.

**Table S8. Subgroup analyses and tests for interaction**

| **Variable** | **Cases** | **High PNI trajectory**  **OR (95% CI)** | **P for interaction** | **Low PNI trajectory**  **OR (95% CI)** | **P for interaction** |
| --- | --- | --- | --- | --- | --- |
| Age |  |  | 0.8428 |  | 0.9435 |
| ＜60 years | 242 | 0.83 (0.46, 1.47) |  | 0.52 (0.20, 1.31) |  |
| ≥60 years | 69 | 0.71 (0.17, 2.89) |  | 0.49 (0.12, 1.95) |  |
| Sex |  |  | 0.4813 |  | 0.9398 |
| Male | 188 | 0.97 (0.50, 1.86) |  | 0.51 (0.20, 1.32) |  |
| Female | 123 | 0.65 (0.27, 1.59) |  | 0.48 (0.13, 1.76) |  |
| Hypertension |  |  | 0.3559 |  | 0.8937 |
| Yes | 275 | 0.85 (0.48, 1.50) |  | 0.59 (0.26, 1.34) |  |
| No | 20 | 0.33 (0.05, 2.37) |  | 0.71 (0.05, 9.50) |  |
| Diabetes |  |  | 0.9652 |  | 0.4838 |
| Yes | 111 | 0.69 (0.21, 2.25) |  | 0.91 (0.30, 2.76) |  |
| No | 184 | 0.67 (0.35, 1.26) |  | 0.52 (0.17, 1.61) |  |
| Renal function |  |  | 0.3969 |  | 0.8714 |
| Low | 150 | 0.62 (0.26, 1.47) |  | 0.47 (0.13, 1.68) |  |
| High | 151 | 1.00 (0.49, 2.04) |  | 0.53 (0.20, 1.42) |  |

Renal function is classified based on the median value of eGFR.

**Table S9.** Sensitivity analysis of the association between PNI trajectories and risk of all-cause mortality, excluding patients who died within the first 12 months

| **Exposure** | **Model 1**  **HR (95%CI)** | ***P*** | **Model 2**  **HR (95%CI)** | ***P*** | **Model 3**  **HR (95%CI)** | ***P*** |
| --- | --- | --- | --- | --- | --- | --- |
| Low-stable group | 1.00 (ref) |  | 1.00 (ref) |  | 1.00 (ref) |  |
| Moderate-stable group | 0.39 (0.23, 0.66) | <0.001 | 0.46 (0.25, 0.83) | 0.0096 | 0.51 (0.24, 1.12) | 0.0941 |
| High-stable group | 0.22 (0.12, 0.41) | <0.001 | 0.23 (0.12, 0.46) | <0.001 | 0.19 (0.08, 0.48) | <0.001 |

Model 1: Adjusted for sex and age.
Model 2: Model 1 additionally adjusted for comorbidities (hypertension, diabetes, heart disease, stroke), smoking, alcohol consumption, and eGFR.
Model 3: Model 2 additionally adjusted for medication use (ARB/ACEI, CCB, diuretics, α/β-blockers), IPTH, Total Kt/V, and BMI.

**Table S10.** Linear mixed-effects model for longitudinal PNI after multiple imputation for missing data

| **Variable** | **β (95% CI）** | **SE** | ***P*** |
| --- | --- | --- | --- |
| PNI |  |  |  |
| Full-dose PD | 0(Reference) | - | - |
| IPD | 0.290 (-1.101, 1.685) | 0.711 | 0.683 |
| Time, months | -0.042 (-0.066, -0.017) | 0.013 | <0.001 |
| Dialysis modality×time |  |  |  |
| Full-dose PD | 0(Reference) | - | - |
| IPD | -0.022 (-0.074, 0.030) | 0.026 | 0.406 |

The model was adjusted for baseline covariates, including sex, age, comorbidities (hypertension, diabetes, cardiovascular disease, stroke), smoking, alcohol consumption, eGFR, medication use (ARB/ACEI, calcium channel blockers, diuretics, α/β-blockers), IPTH, total Kt/V, and BMI.

**Table S11. Sensitivity analysis of the association between initial dialysis modality and PNI trajectory membership using multiple imputation**

| **Exposure** | **Model 1**  **OR (95%CI)** | ***P*** | **Model 2**  **OR (95%CI)** | ***P*** | **Model 3**  **OR (95%CI)** | ***P*** |
| --- | --- | --- | --- | --- | --- | --- |
| High PNI trajectory |  |  |  |  |  |  |
| Full-dose PD | 1.00 (ref) |  | 1.00 (ref) |  | 1.00 (ref) |  |
| IPD | 0.77 (0.45, 1.34) | 0.356 | 0.66 (0.37, 1.19) | 0.169 | 0.80 (0.44, 1.47) | 0.471 |
| Low PNI trajectory |  |  |  |  |  |  |
| Full-dose PD | 1.00 (ref) |  | 1.00 (ref) |  | 1.00 (ref) |  |
| IPD | 0.51 (0.24, 1.12) | 0.096 | 0.53 (0.23, 1.18) | 0.119 | 0.51 (0.22, 1.19) | 0.118 |

Model 1: Adjusted for sex and age.

Model 2: Model 1 additionally adjusted for comorbidities (hypertension, diabetes, heart disease, stroke), smoking, alcohol consumption, and eGFR.

Model 3: Model 2 additionally adjusted for medication use (ARB/ACEI, CCB, diuretics, α/β-blockers), IPTH, total Kt/V, and BMI.

**Table S12.** Covariate balance before and after entropy balancing-derived inverse probability weighting

| **Variables** | **Unweighted ASMD** | **Weighted ASMD** |
| --- | --- | --- |
| BMI | 0.261 | <0.001 |
| eGFR | 0.249 | <0.001 |
| iPTH | 0.188 | <0.001 |
| Diabetes mellitus | 0.143 | <0.001 |
| ACEI/ARB | 0.141 | <0.001 |
| Age | 0.106 | <0.001 |
| CCB | 0.101 | <0.001 |
| Sex | 0.067 | <0.001 |
| Total Kt/V | 0.063 | <0.001 |
| Smoking status | 0.056 | <0.001 |
| Hypertension | 0.054 | <0.001 |
| Alcohol consumption | 0.052 | <0.001 |
| Heart disease | 0.051 | <0.001 |
| α/β-blockers | 0.025 | <0.001 |
| Diuretics | 0.022 | <0.001 |
| Stroke | 0.021 | <0.001 |

**Table S13. Sensitivity analysis of the association between initial dialysis modality and PNI trajectory membership using inverse probability weighted multivariable logistic regression with multiple imputation**

| **Exposure** | **Model 1**  **OR (95%CI)** | ***P*** | **Model 2**  **OR (95%CI)** | ***P*** | **Model 3**  **OR (95%CI)** | ***P*** |
| --- | --- | --- | --- | --- | --- | --- |
| High PNI trajectory |  |  |  |  |  |  |
| Full-dose PD | 1.00 (ref) | - | 1.00 (ref) | - | 1.00 (ref) | - |
| IPD | 0.70 (0.36, 1.37) | 0.295 | 0.69 (0.34, 1.39) | 0.296 | 0.68 (0.33, 1.38) | 0.287 |
| Low PNI trajectory |  |  |  |  |  |  |
| Full-dose PD | 1.00 (ref) | - | 1.00 (ref) | - | 1.00 (ref) | - |
| IPD | 0.61 (0.25, 1.49) | 0.276 | 0.59 (0.25, 1.40) | 0.229 | 0.58 (0.24, 1.38) | 0.213 |

Model 1: Adjusted for sex and age.

Model 2: Model 1 additionally adjusted for comorbidities (hypertension, diabetes, heart disease, stroke), smoking, alcohol consumption, and eGFR.

Model 3: Model 2 additionally adjusted for medication use (ARB/ACEI, CCB, diuretics, α/β-blockers), IPTH, total Kt/V, and BMI.

**Table S14. Causal mediation analysis of baseline RKF (eGFR) on the association between initial dialysis modality and PNI trajectories**

| **Effect** | **Standard Estimate (95% CI)** | **P value** | **Bootstrap Estimate (95% CI)** | **P value** |
| --- | --- | --- | --- | --- |
| **High PNI trajectory** |  |  |  |  |
| Average Causal Mediation Effects (ACME) | -0.0082 (-0.0291, 0.0059) | 0.270 | -0.0084 (-0.0255, 0.0071) | 0.302 |
| Average Direct Effects (ADE) | -0.0368 (-0.1579, 0.0930) | 0.544 | -0.0360 (-0.1539, 0.0868) | 0.556 |
| Total Effect | -0.0450 (-0.1679, 0.0814) | 0.480 | -0.0444 (-0.1624, 0.0772) | 0.482 |
| Proportion of Mediated | 0.0574 (-1.4858, 1.3120) | 0.630 | 0.1891 (-1.1943, 1.5884) | 0.656 |
| **Low PNI trajectory** |  |  |  |  |
| Average Causal Mediation Effects (ACME) | 0.0087 (-0.0012, 0.0257) | 0.110 | 0.0087 (-0.0033, 0.0238) | 0.166 |
| Average Direct Effects (ADE) | -0.0922 (-0.1766, 0.0088) | 0.068 | -0.0953 (-0.1813, -0.0006) | 0.050 |
| Total Effect | -0.0835 (-0.1695, 0.0181) | 0.100 | -0.0867 (-0.1731, 0.0066) | 0.070 |
| Proportion of Mediated | -0.0771 (-1.0620, 0.5344) | 0.202 | -0.1000 (-0.8283, 0.4328) | 0.216 |

**Table S15.** Sensitivity analysis of the association between PNI trajectories and risk of all-cause mortality adjusting for baseline CRP

| **Exposure** | **Fully Adjusted HR (95% CI)** | **P** |
| --- | --- | --- |
| Low-stable group | 1.00 (ref) | - |
| Moderate-stable group | 0.37 (0.21, 0.67) | 0.001 |
| High-stable group | 0.19 (0.10, 0.38) | <0.001 |

**Table S16.** Sensitivity analysis of the association between initial dialysis modality and PNI trajectory membership adjusting for baseline CRP

| **Exposure** | **Fully Adjusted OR (95%CI)** | **P** |
| --- | --- | --- |
| High PNI trajectory |  |  |
| Full-dose PD | 1.00 (ref) | - |
| IPD | 0.82 (0.44, 1.52) | 0.523 |
| Low PNI trajectory |  |  |
| Full-dose PD | 1.00 (ref) | - |
| IPD | 0.43 (0.17, 1.06) | 0.067 |

**Table S17.** Sensitivity analysis of the association between PNI trajectories and all-cause mortality, additionally adjusted for PDAP

| **Exposure** | **Adjusted HR (95% CI)** | **P value** |
| --- | --- | --- |
| Low-stable group | 1.00 (Reference) |  |
| Moderate-stable group | 0.38 (0.21-0.69) | 0.002 |
| High-stable group | 0.20 (0.10-0.39) | <0.001 |

Notes: This model builds upon the fully adjusted Model 3 by additionally incorporating the occurrence of peritoneal dialysis-associated peritonitis (PDAP) as a covariate. The final adjustments include sex, age, comorbidities (hypertension, diabetes, heart disease, stroke), smoking, alcohol consumption, baseline eGFR, medication use (ARB/ACEI, CCB, diuretics, α/β-blockers), iPTH, total Kt/V, BMI, and PDAP.

**Table S18.** Sensitivity analysis of the association between initial PD modality and PNI trajectories, additionally adjusted for PDAP

| **Exposure** | **Adjusted OR (95% CI)** | **P value** |
| --- | --- | --- |
| **High PNI trajectory** |  |  |
| Full-dose PD | 1.00 (Reference) |  |
| IPD | 0.71 (0.38-1.34) | 0.298 |
| **Low PNI trajectory** |  |  |
| Full-dose PD | 1.00 (Reference) |  |
| IPD | 0.51 (0.21-1.21) | 0.127 |

Notes: This model builds upon the fully adjusted multivariable logistic regression (Model 3) by additionally incorporating the occurrence of peritoneal dialysis-associated peritonitis (PDAP) as a covariate. The final adjustments include sex, age, comorbidities (hypertension, diabetes, heart disease, stroke), smoking, alcohol consumption, baseline eGFR, medication use (ARB/ACEI, CCB, diuretics, α/β-blockers), iPTH, total Kt/V, BMI, and PDAP.

**Table S19.** Step-wise sensitivity analysis evaluating the prognostic value of baseline PNI and longitudinal PNI trajectories for all-cause mortality

| **Analytical Model** | **Exposure Variable** | **Adjusted HR (95% CI)** | **P value** |
| --- | --- | --- | --- |
| **Model A: Baseline PNI Model** | Baseline PNI (continuous) | 0.96 (0.93–1.00) | 0.073 |
| **Model B: Trajectory Model** | Low-stable group | 1.00 (Reference) | - |
|  | Moderate-stable group | 0.45 (0.22–0.92) | 0.028 |
|  | High-stable group | 0.17 (0.07–0.40) | <0.001 |
| **Model C: Combined Model** | Baseline PNI (continuous) | 1.00 (0.95–1.04) | 0.897 |
|  | Low-stable group | 1.00 (Reference) | - |
|  | Moderate-stable group | 0.38 (0.21–0.69) | 0.002 |
|  | High-stable group | 0.21 (0.10–0.43) | <0.001 |

Notes: Hazard ratios were derived from multivariable Cox proportional hazards regression models.

Model A evaluates the continuous baseline PNI alone.

Model B evaluates the categorical PNI trajectory alone (results correspond to Model 3 in the main text).

Model C incorporates both baseline PNI and the categorical PNI trajectory simultaneously to assess additive prognostic value.

All three models were adjusted for an identical set of baseline covariates: sex, age, comorbidities (hypertension, diabetes, heart disease, stroke), smoking, alcohol consumption, BMI, medication use (ARB/ACEI, CCB, diuretics, α/β-blockers), eGFR, iPTH, and total Kt/V.

**Table S20.** E-value analysis to assess the robustness of the association between PNI trajectories and all-cause mortality to unmeasured confounding

| **Exposure** | **Effect Measure (RR scale)** | **95% CI** | **E-value for Point Estimate** | **E-value for Confidence Interval** |
| --- | --- | --- | --- | --- |
| Low-stable group | 1.00 (ref) | - | - | - |
| Moderate-stable group | 1.73 | 1.06, 2.78 | 2.86 | 1.31 |
| High-stable group | 3.27 | 1.88, 5.53 | 6.00 | 3.16 |

**Table S21.** E-value analysis to assess the robustness of the association between initial dialysis modality and PNI trajectory membership to unmeasured confounding

| **Outcome** | **Effect Measure (RR scale)** | **95% CI** | **E-value for Point Estimate** | **E-value for Confidence Interval** |
| --- | --- | --- | --- | --- |
| High PNI trajectory | 1.18 | 0.83, 1.67 | 1.64 | 1.00 |
| Low PNI trajectory | 1.31 | 0.76, 2.24 | 1.95 | 1.00 |

**Figure S1.** Scaled Schoenfeld residual plot for the PNI trajectory.

**Figure S2.** Covariate balance before and after inverse probability weighting
